# Effects of a Multidisciplinary Intervention on Daily-Living Gait Among Older Adults With Parkinson’s Disease

**DOI:** 10.1093/geroni/igaa057.746

**Published:** 2020-12-16

**Authors:** Jeffrey Hausdorff, Moriya Cohen, Natalie Ganz, Yitchak Green, Inbal Badichi, Tanya Curevich

**Affiliations:** 1 Tel Aviv Sourasky Medical Center, Tel Aviv, Tel Aviv, Israel; 2 EZRA–LEMARPE, Bnei Bar, Israel; 3 Tel Aviv Sourasky Medical Center, Tel Aviv, Israel; 4 EZRA–LEMARPE Organization, Bnei Brak, Israel; 5 EZRA–LEMARPE Orginization, Bnei Brak, Israel

## Abstract

Multidisciplinary interventions can improve gait and balance in patients with Parkinson’s disease (PD). However, it is not yet known if these interventions also positively impact the quality of daily-living walking. We, therefore, examined the effects of a multidisciplinary, intensive out-patient rehabilitation program (MIOR) as delivered by the rehabilitation center of EZRA–LEMARPE organization on gait and balance as measured in the clinic and on every-day walking, as measured during 1-week of continuous measurement. 46 PD patients (age: 70.05±7.71; gender: 31.3% women; disease duration: 8.85±6.27 yrs) were evaluated before and after participating in 8-weeks of physical, occupational, and hydro-therapy, boxing, and dance (3 days/week; 5 hrs/day). After the intervention, clinical measures of balance (MiniBest Test of Balance delta: 1.82±3.30 points, p=0.001), mobility (TUG delta: -1.78±6.15sec; p=0.001), and usual-walking speed (delta 19±16cm/s; p<0.001) improved. Daily-living step counts and daily-living gait quality did not change (p>0.5). In exploratory analyses, subjects were categorized as responders (Rs) and non-responders (NRs) based on changes in their daily-living walking gait speed. Rs increased their daily-living gait speed (delta: 10±14cm/s; p<0.001); NRs did not. Rs (n=21) also improved their daily-living gait quality measures (e.g. stride regularity, step length, stride time variability). At baseline, disease severity (MDS-UPDRSIII) was lower (p=0.02) in Rs (25.33±11.47), compared to the NRs (34.38±14.27). These results demonstrate that improvements in the clinic do not necessarily transfer to improvements in daily-living gait. Further, in select patients, MIOR can ameliorate daily-living walking quality, potentially reducing the risk of falls and other adverse outcomes associated with impaired mobility.

